# Effects of Poly-Si Grain Boundary on Retention Characteristics under Cross-Temperature Conditions in 3-D NAND Flash Memory

**DOI:** 10.3390/mi14122199

**Published:** 2023-11-30

**Authors:** Ukju An, Gilsang Yoon, Donghyun Go, Jounghun Park, Donghwi Kim, Jongwoo Kim, Jeong-Soo Lee

**Affiliations:** Department of Electrical Engineering, Pohang University of Science and Technology, Pohang 37673, Republic of Korea; ukjuan@postech.ac.kr (U.A.); ygs6233@postech.ac.kr (G.Y.); godhyun@postech.ac.kr (D.G.); mancity@postech.ac.kr (J.P.); kdh3879@postech.ac.kr (D.K.); jwkim0115@postech.ac.kr (J.K.)

**Keywords:** 3-D NAND flash memory, cross-temperature, threshold voltage variation, decomposition, poly-Si channel, grain boundary, charge loss

## Abstract

Electrical characteristics with various program temperatures (*T_PGM_*) in three-dimensional (3-D) NAND flash memory are investigated. The cross-temperature conditions of the *T_PGM_* up to 120 °C and the read temperature (*T_READ_*) at 30 °C are used to analyze the influence of grain boundaries (GB) on the bit line current (*I_BL_*) and threshold voltage (*V_T_*). The *V_T_* shift in the E-P-E pattern is successfully decomposed into the charge loss (Δ*V_T,CL_*) component and the poly-Si GB (Δ*V_T__,GB_*) component. The extracted Δ*V_T__,GB_* increases at higher *T_PGM_* due to the reduced GB potential barrier. Additionally, the Δ*V_T__,GB_* is evaluated using the Technology Computer Aided Design (TCAD) simulation, depending on the GB position (*X_GB_*) and the bit line voltage (*V_BL_*).

## 1. Introduction

As NAND flash memory is widely used in smartphones and data centers, the demand for high-density, low-cost NAND flash memory continues to surge. Due to the limitations in scaling, the conventional planar two-dimensional (2-D) NAND has been rapidly substituted with three-dimensional (3-D) NAND [1,2]. In 3-D NAND, the vertical stacking of cells is less constrained by the scaling challenges, effectively boosting the storage capacity. Furthermore, multi-level cell technology has been adopted to further enhance the storage capacity and has evolved to the point of 4-bits/cell (quadruple level cell, QLC) development [3]. However, as the number of bits per cell increases, the threshold voltage (*V_T_*) window narrows, necessitating the tight *V_T_* distribution. A slight change in *V_T_* can cause a fail bit, which is greatly affected by the environmental temperature.

NAND flash memory operates within a temperature range typically from 0 °C to 85 °C. However, such as in automotive or aerospace applications, NAND flash memory can be designed to function within a broader temperature range, extending from −40 °C to 125 °C [4,5]. The NAND operations, such as program and read, need to be executed precisely within the prescribed temperature range, even if the temperatures vary during operations. The temperature change can significantly impact both the trapped charge within the nitride layer and the electrical characteristics of the channel [6,7,8]. The utilization of SiN material as a charge storage layer causes various charge loss mechanisms and *V_T_* changes during the retention in NAND flash memory. At elevated temperatures, the thermal emission of trapped electrons accelerates, consequently increasing the amount of de-trapping from the charge trap layer (CTL) to the channel. Additionally, the continuous CTL in the 3-D NAND induces additional charge migration in the lateral direction. This lateral migration is dominantly caused by Poole-Frenkel emission and becomes more pronounced at higher temperatures [9,10]. These charge loss mechanisms can change the *V_T_* and potentially influence the reliability of the NAND devices in response to temperature variations.

Three-dimensional NAND is fabricated in the following process. The multi-stacked SiN/oxide layers are deposited. The memory hole is formed through the etching process, and it is then filled by the ONO layer and channel layer deposition. Subsequently, the SiN layers are removed, and empty spaces are filled by gate stack deposition [11]. The poly-Si, which can be easily formed through the deposition process, is used as a channel material in 3-D NAND due to the difficulty of single crystalline silicon growth in the process sequence. In the poly-Si deposition process, amorphous silicon is deposited and annealed to form grains [12]. Therefore, the poly-Si channel contains grain boundaries (GBs) between the Si grains. Depending on the bias conditions, carriers can be trapped at these GBs [13]. The GB trapped charges form the potential barrier (*Φ_B_*) and interfere with carrier transport [14,15,16,17]. An elevated temperature can decrease the trap occupancy, lowering the *Φ_B_* of GB compared with room temperature [18,19]. Consequently, it leads to low *V_T_* and high bit line current (*I_BL_*) even though the channel mobility is degraded [8]. Hence, when the temperature changes during the NAND operations, the temperature-induced *V_T_* variation attributed to charge loss and poly-Si GB should be considered.

The cross-temperature is the condition where the program temperature (*T_PGM_*) differs from the subsequent read temperature (*T_READ_*). Additional fail bits during read operation occur in cross-temperature conditions. There are many studies on controlling additional fail bits in the cross-temperature conditions at the system level. The most common methods are to use the temperature compensation circuits, which track temperature changes through sensors and then change the read reference voltages [20,21]. However, these compensation methods struggle to address all fail bits in cross-temperature conditions [22,23]. The relevant temperature coefficient in the compensation circuit differs among individual cells [24]. Even in the same cell, this coefficient varies depending on the temperature conditions: a high temperature program and low temperature read (HPLR) condition or a low temperature program and high temperature read (LPHR) condition [25,26]. Therefore, more detailed analyses at the cell level are needed to understand the cross-temperature phenomena and effectively reduce the fail bits in 3-D NAND.

Here, the retention characteristics of a programmed cell under the HPLR conditions are characterized and decomposed into the charge loss and poly-Si GB components using a neutral cell. The poly-Si GB components are further investigated with different bit line voltages (*V_BL_*). Technology Computer Aided Design (TCAD) simulation is conducted to understand the relationship between the *V_BL_* and the GB position.

## 2. Results and Discussion

### 2.1. Cross-Temperature Effects on Retention Characteristics

**Figure 1:**
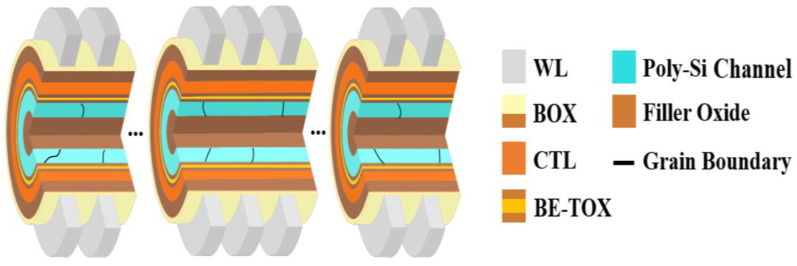
A schematic of the 3-D NAND flash memory used in this work. The filler oxide, poly-Si channel, bandgap-engineered tunneling oxide (BE-TOX), charge trap layer (CTL), blocking oxide with high-κ material (BOX), and word line (WL) are indicated. Randomly distributed grains and grain boundaries are indicated in the poly-Si channel.

Figure 1 shows the schematic of the 3-D NAND flash memory with 24 stacked word line (WL) layers used in this experiment. It is composed of the filler oxide, poly-Si channel, bandgap-engineered tunneling oxide (BE-TOX) consisting of O1/N1/O2 layers, CTL, blocking oxide with high-κ material (BOX), and WL. Each WL is separated by the spacer. In contrast to the 2-D planar NAND structure, a poly-Si channel with randomly distributed multiple grains is used. Each grain forms GBs, where channel carriers can be trapped. The *Φ_B_* of the GB is formed due to the trapped charges at the GB and interferes with carrier transport. Both ends of the channel are connected by the source line (SL) and bit line (BL).

**Figure 2:**
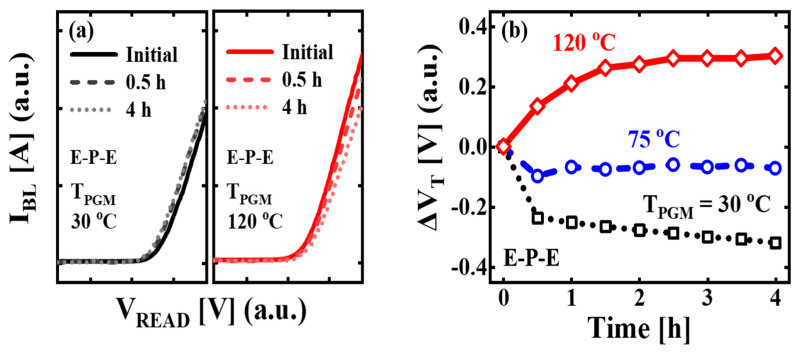
(**a**) The time-dependent bit-line current (*I_BL_*) vs. read voltage (*V_READ_*) characteristics under the E-P-E pattern at retention time of initial (=30 s), 0.5 h, and 4 h. The program temperatures (*T_PGM_*) are 30 °C and 120 °C, respectively. (**b**) The *V_T_* shift (Δ*V_T_*) in the target programmed cell under the E-P-E pattern at *T_PGM_* = 30 °C, 75 °C, and 120 °C. The cases of *T_PGM_* = 75 °C and *T_PGM_* = 120 °C are the high temperature program and low temperature read (HPLR) conditions. The read temperature (*T_READ_*) after 4 h is 30 °C.

Figure 2a shows the time-dependent *I_BL_* versus read voltage (*V_READ_*) characteristics at *T_PGM_* = 30 °C and 120 °C. For the read operation, *V_READ_* was applied to the target cell as the word line voltage, and pass voltage was applied to the rest of the cells except the target cell. The *V_BL_* was applied with 1 V. The retention characteristics were measured for the E-P-E pattern, with the 7th-program verifying (PV7) level. All cells in the string were initially erased to eliminate the residual electrons in CTL to prevent any additional charge loss by electrons of the neighbor cell, and only the target cell was programmed. The initial read operation was performed at 30 s immediately after the program operation. All cases of *T_PGM_* > 30 °C are HPLR conditions. The *T_READ_* was promptly lowered to 30 °C through cooling after the program operation in HPLR conditions. This means that the initial *T_READ_* is the same as *T_PGM_*, and the *T_READ_* gradually decreases over time. The final *T_READ_* after 4 h is 30 °C. For *T_PGM_* = 30 °C, the *I_BL_*-*V_READ_* curve shifts towards the left direction over time. For *T_PGM_* = 120 °C, the *I_BL_*-*V_READ_* curve shifts towards the right direction over time. Figure 2b shows the retention characteristics at *T_PGM_* = 30 °C, 75 °C, and 120 °C. In all cases, the *T_READ_* after 4 h is 30 °C. The *V_T_* was extracted using the constant current method at *I_BL_* = 1 µA. The Δ*V_T_* was calculated by subtracting the initial *V_T_* from the *V_T_* at each retention time. The negative Δ*V_T_* is observed at *T_PGM_* = 30 °C, indicating the decrease in *V_T_* over time. For *T_PGM_* = 75 °C, the Δ*V_T_* negligibly changes over time, even though trapped electrons are emitted from CTL to the channel in the vertical direction or spread from the gate to spacer in the lateral direction during the retention. For *T_PGM_* = 120 °C, the positive Δ*V_T_* over time is observed up to 4 h.

**Figure 3:**
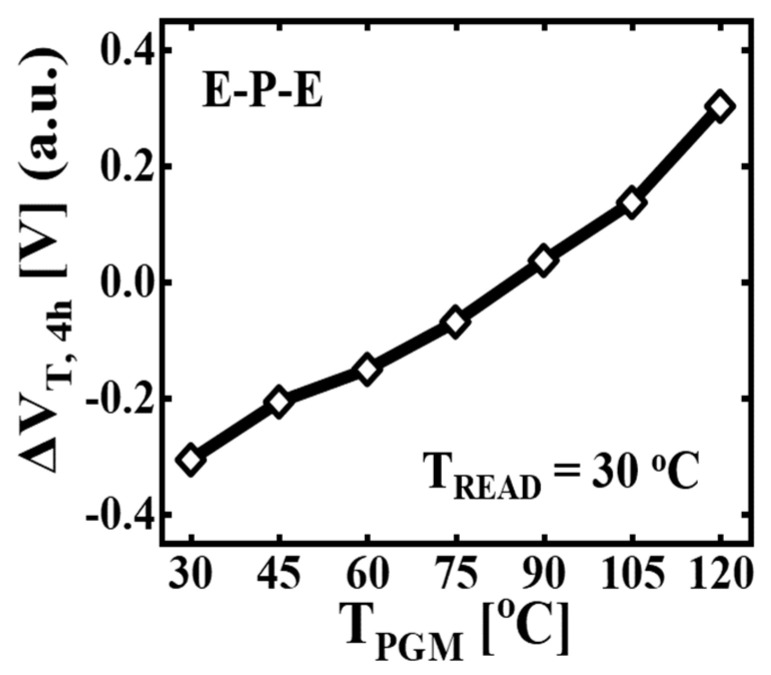
The Δ*V_T_* after 4 h (Δ*V_T,4h_*) with different *T_PGM_* under E-P-E pattern, ranging from 30 °C to 120 °C. All cases of *T_PGM_* > 30 °C are HPLR conditions. The *T_READ_* after 4 h is 30 °C.

Figure 3 shows the Δ*V_T_* after 4 h (Δ*V_T,4h_*) with respect to *T_PGM_*. All cases except *T_PGM_* = 30 °C are HPLR conditions. For all *T_PGM_* values ranging from 30 °C to 120 °C, the *T_READ_* after 4 h is 30 °C. The Δ*V_T,4h_* increases linearly with *T_PGM_* up to 120 °C. For *T_PGM_* = 30 °C, the negative Δ*V_T,4h_* is observed due to the Δ*V_T_* by charge loss (Δ*V_T,CL_*). At the elevated *T_PGM_*, the Δ*V_T,4h_* increases due to the Δ*V_T_* by poly-Si GB (Δ*V_T,GB_*). Since Δ*V_T,CL_* has a negative value at elevated *T_PGM_*, the increase in Δ*V_T,4h_* by *T_PGM_* indicates that the influence of Δ*V_T,GB_* on Δ*V_T,4h_* is higher than that of Δ*V_T,CL_* at elevated *T_PGM_*.

**Figure 4:**
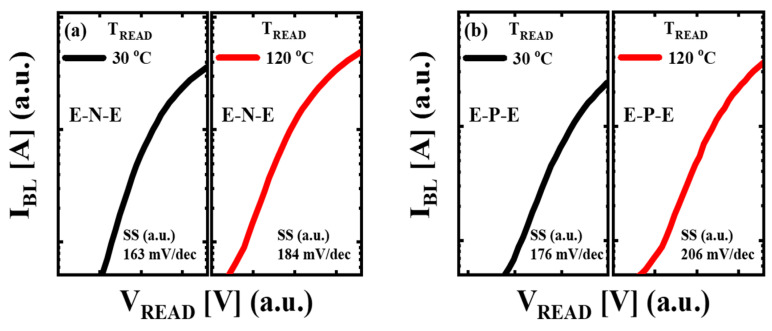
The *I_BL_*-*V_READ_* characteristics at *T_READ_* = 30 °C and 120 °C for the (**a**) E-N-E and (**b**) E-P-E patterns. The subthreshold swing (*SS*) of E-N-E and E-P-E patterns under *T_READ_* = 30 °C and 120 °C is indicated. The *SS* is higher at *T_READ_* = 120 °C than at *T_READ_* = 30 °C for both the E-N-E and E-P-E patterns. The *SS* difference value of the E-P-E pattern between *T_READ_* = 30 °C and *T_READ_* = 120 °C is higher than that of the E-N-E pattern.

To decompose the Δ*V_T_* of the target cell under the E-P-E pattern into Δ*V_T,GB_* and Δ*V_T,CL_* components, the Δ*V_T,GB_* is primarily extracted by using the E-N-E pattern. The E-N-E pattern is adopted to suppress the charge loss of the target cell in CTL, which is obtained by erasing all cells and soft programming the target cell up to the *V_T_* of the fresh cell. The Δ*V_T,GB_* of E-N-E pattern (Δ*V_T,GBN_*) between *T_READ_* = 30 °C and *T_READ_* = *T* can be obtained by the following equation,

(1) $$\Delta V_{T,GBN}=V_{T,N}(30\;{}^{\circ}C)-V_{T,N}(T)$$

where *V_T,N_* (*T*) is the *V_T_* of the E-N-E pattern at *T_READ_* = *T*. The neutral cell has no trapped charges in CTL, which causes negligible Δ*V_T,CL_* during retention [6,27]. Figure 4a shows the *I_BL_*-*V_READ_* curves at *T_READ_* = 30 °C and 120 °C for the E-N-E pattern. The subthreshold swing (*SS*) is higher at *T_READ_* = 120 °C compared to *T_READ_* = 30 °C. Figure 4b shows the *I_BL_*-*V_READ_* curves at *T_READ_* = 30 °C and 120 °C for the E-P-E pattern. The *SS* is also higher at *T_READ_* = 120 °C than at *T_READ_* = 30 °C. The *SS* difference in the E-N-E pattern between *T_READ_* = 30 °C and *T_READ_* = 120 °C is lower than that in the E-P-E pattern.

**Figure 5:**
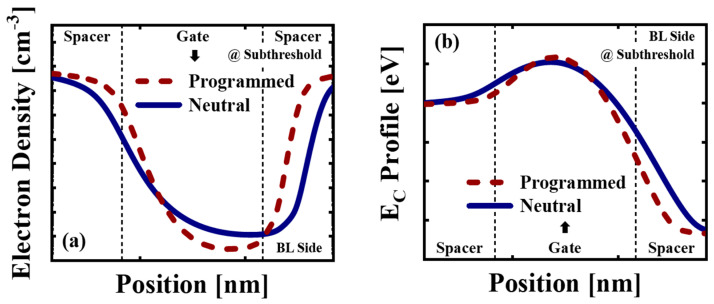
(**a**) The channel electron densities along the bit line (BL) direction for the neutral and programmed target cells using Technology Computer Aided Design (TCAD) simulations. The electron densities are obtained near the BE-TOX/channel interface at the subthreshold region under bit line voltage (*V_BL_*) = 1 V. (**b**) The energy band diagrams (*E_C_*) near the BE-TOX/channel interface along BL direction for the neutral and programmed target cells.

The *SS* differences in the E-N-E and E-P-E patterns are analyzed using TCAD simulation [28]. The string of 3-D NAND used in the TCAD simulation consisted of a source select line, a drain select line, two dummy cells on both sides, and five word lines to minimize the simulation time. Except for the number of WLs, all dimension parameters used to construct the simulation structure were the same as the actual device dimension parameters. For the electron and hole traps in CTL, the Gaussian energy distributions were used. Hurkx band-to-band tunneling model for gate induced drain leakage (GIDL) erase, and the Shockley-Read-Hall (SRH) model for capture or emission of carriers into traps were adopted. Transient simulation with the non-local tunneling model was applied for program and erase operations [29]. Figure 5a shows the channel electron density near the BE-TOX interface along the BL direction for the neutral and programmed target cells at the subthreshold region. The *V_BL_* is applied with 1 V. For the programmed cell, the trapped charge density in the CTL is higher at the middle of the gate region than at the gate edge. The channel electron density of the programmed cell is higher than that of the neutral cell at the edge region. Figure 5b shows the conduction energy band (*E_C_*) profile in the channel near the BE-TOX interface along the BL direction for the neutral and programmed target cells at the subthreshold region. For the programmed cell, the *E_C_* peak is higher than the neutral cell and the effective channel length decreases due to the nonuniform trapped charge distribution in the CTL [30]. As the effective channel length is reduced, the influence of GB decreases and the *SS* difference in the E-P-E pattern between different *T_READ_* is higher than the *SS* difference in the E-N-E pattern [8]. Therefore, the Δ*V_T,GB_* of the E-P-E pattern is lower than the Δ*V_T,GBN_*. The Δ*V_T,GB_* of the E-P-E pattern can be obtained using the Δ*V_T,GBN_* with the following equation,

(2) $$\Delta V_{T,GB}=\Delta V_{T,GBN}-V_{SS}$$

where *V_SS_* value is the *SS* difference correction voltage, which is defined using the threshold current (*I_T_*) at *V_READ_* = *V_T_* and off-current (*I_OFF_*) as follows:

(3) $$V_{SS}=\left(\Delta {SS}_{P}-\Delta {SS}_{N}\right)\times \log_{10}\left(I_{T}/I_{OFF}\right)$$

where Δ*SS_P_* and Δ*SS_N_* are the *SS* difference values between *T_READ_* = 30 °C and *T_READ_* = *T* for the E-P-E and E-N-E patterns, respectively.

**Figure 6:**
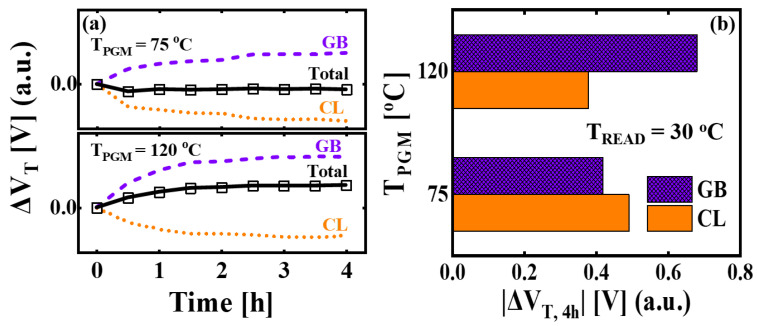
(**a**) The decomposed retention characteristics of measured Δ*V_T_* into Δ*V_T_* by poly-Si GB (Δ*V_T,GB_)* and Δ*V_T_* by charge loss (Δ*V_T,CL_)* components under the E-P-E pattern for *T_PGM_* = 75 °C and 120 °C. (**b**) The absolute values of Δ*V_T,4h_* for decomposed GB and CL components with different *T_PGM_* (= 75 °C and 120 °C). The *T_READ_* after 4 h is 30 °C.

Figure 6a shows the decomposed results of Δ*V_T_* into Δ*V_T,GB_* and Δ*V_T,CL_* for *T_PGM_* = 75 °C and 120 °C. The charge loss components are obtained by subtracting the GB components from Δ*V_T_* of the E-P-E pattern, which is expressed as follows:

(4) $$\Delta V_{T,CL}=\Delta V_{T,TOTAL}-\Delta V_{T,GB}$$

where Δ*V_T,TOTAL_* is the measured Δ*V_T_* of the E-P-E pattern. The Δ*V_T,GB_* increases during the retention time for both *T_PGM_* = 75 °C and 120 °C. The Δ*V_T,CL_* has a negative value up to 4 h. Figure 6b shows the absolute values of the decomposed Δ*V_T,4h_* at *T_PGM_* = 75 °C and 120 °C. The Δ*V_T,CL_* diminishes at the elevated *T_PGM_* due to the fact that the higher *T_PGM_* results in more significant charge loss within the initial 30 s, corresponding to the range of short-term retention [26,31,32]. The Δ*V_T,GB_* increases at the elevated *T_PGM_* due to the decrease in *Φ_B_* of the GB at higher temperatures. The Δ*V_T,GB_* is 15% lower than the Δ*V_T,CL_* at *T_PGM_* = 75 °C, and the Δ*V_T,CL_* is 45% lower than the Δ*V_T,GB_* at *T_PGM_* = 120 °C. With an increase in *T_PGM_* under the HPLR condition, the influence of Δ*V_T,GB_* on Δ*V_T,4h_* becomes more pronounced.

### 2.2. Threshold Voltage Shift by Poly-Si GB

**Figure 7:**
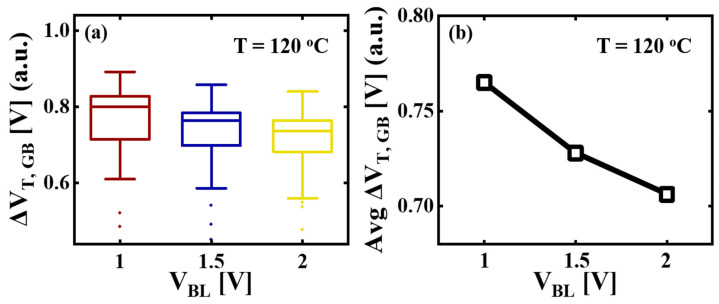
(**a**) The Δ*V_T,GB_* of 50 neutral cells for *T* = 120 °C at *V_BL_* = 1 V, 1.5 V, and 2 V. The Δ*V_T,GB_* is the *V_T_* difference between *T_READ_* = 30 °C and *T_READ_* = *T*. The box indicates the data set of Δ*V_T,GB_* ranging from 25% to 75% with the horizontal line, which is the median. The boundaries of the whiskers are minimum and maximum Δ*V_T,GB_*, which are the lowest data and highest data excluding any outliers. The single points on the diagram show the outliers. (**b**) The average (Avg) value of Δ*V_T,GB_* for *T* = 120 °C at *V_BL_* = 1 V, 1.5 V, and 2 V.

Figure 7a shows the Δ*V_T,GB_* observed in 50 cells as a function of *V_BL_*. In order to exclude the influence of differences in memory hole diameter that may occur depending on the location of the WL, only the middle five WLs from several different strings were used.

The physical dimensions of the measured cells are all the same. The Δ*V_T,GB_* is the *V_T_* difference between *T_READ_* = 30 °C and *T_READ_* = *T*. The neutral cells were used to evaluate only the Δ*V_T,GB_*. Figure 7b shows the average value of Δ*V_T,GB_* for *T* = 120 °C at each *V_BL_*. The average Δ*V_T,GB_* decreases by 10% with an increase in *V_BL_* from 1 V to 2 V. To further understand the effect of *V_BL_* on the Δ*V_T,GB_*, the *E_C_* profile in the channel was investigated using the TCAD simulation. A single GB was introduced in the channel under the target cell, varying the GB position (*X_GB_*) in the function of gate length (*L_G_*). The edge of the target cell on the SL side was defined as *X_GB_* = 0. The 3-D random Voronoi grain pattern was applied under the remaining channel regions [33,34]. A U-shaped GB trap profile consisting of donor-like states and acceptor-like states was used for each GB [35]. The interface traps were also considered at the BE-TOX/channel and filler oxide/channel interfaces. The constant mobility for the drift/diffusion transport within the grain and the thermionic boundary conditions at GBs were utilized in the channel [17,18].

**Figure 8:**
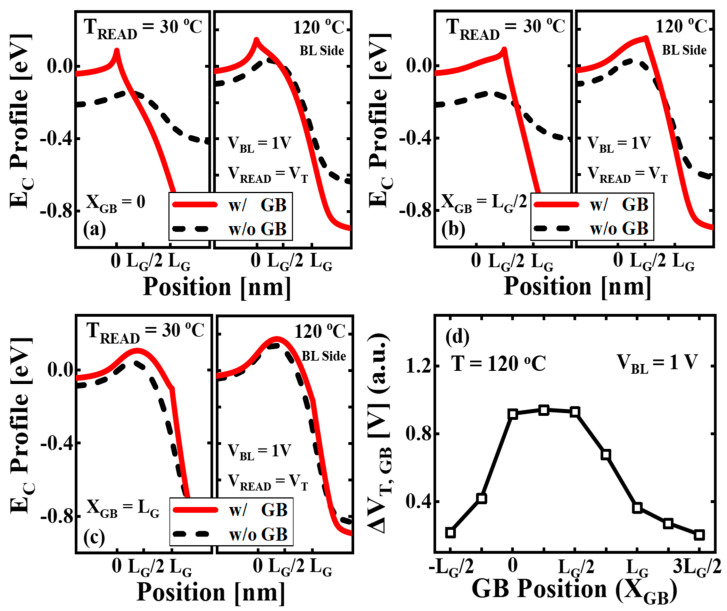
The simulated *E_C_* profiles of the cells with GB (w/ GB) and without GB (w/o GB) along the BL direction at *T_READ_* = 30 °C and 120 °C. The *E_C_* profiles of each cell are obtained at the BE-TOX/channel interface. The *V_READ_* is applied as *V_T_* of the cell with GB and *V_BL_* is fixed as 1 V. The GB position (*X_GB_*) is varied as function of gate length (*L_G_*) under the target cell, which is located at (**a**) *X_GB_* = 0, (**b**) *X_GB_ = L_G_*/2, (**c**) *X_GB_ = L_G_*. (**d**) The simulated Δ*V_T,GB_* depending on each *X_GB_* ranging from -*L_G_*/2 to 3*L_G_*/2 for *T* = 120 °C at *V_BL_* = 1 V.

Figure 8a shows the simulated *E_C_* profiles of the channel along the BL direction for *X_GB_* = 0. The *V_T_* of the cell with GB is applied as *V_READ_* for both cells with GB and without GB. For the cell with GB, the *Φ_B_* induced by GB has an effect as an additional potential barrier. The *Φ_B_* of the cell with GB is higher than that of the cell without GB due to the *Φ_B_* of GB. Figure 8b shows the *E_C_* profiles for *X_GB_* = *L_G_*/2. The *Φ_B_* of the cell with GB is also higher than that of the cell without GB due to the *Φ_B_* of GB. For *X_GB_* = 0 and *L_G_*/2, the *E_C_* difference between the cells with GB and without GB is more significant for *T_READ_* = 30 °C than *T_READ_* = 120 °C. It is due to the increase in *Φ_B_* of GB at lower temperatures. Figure 8c shows the *E_C_* profiles for *X_GB_* = *L_G_*. When GB is located at *X_GB_* = *L_G_*, the *Φ_B_* of GB has negligible effect as the additional potential barrier. The *E_C_* difference between the cells with GB and without GB is reduced at both *T_READ_* = 30 °C and 120 °C due to the decrease in the influence of GB. Figure 8d shows the simulated Δ*V_T,GB_* for *T* = 120 °C at each *X_GB_*. A higher *Φ_B_* is formed at 0 < *X_GB_* < *L_G_*/2, resulting in the substantial Δ*V_T,GB_*. At *L_G_*/2 < *X_GB_* < *L_G_*, the GB effect diminishes and the Δ*V_T,GB_* decreases. When the GB is located under the spacer region, the GB has negligible influence on the Δ*V_T,GB_*.

**Figure 9:**
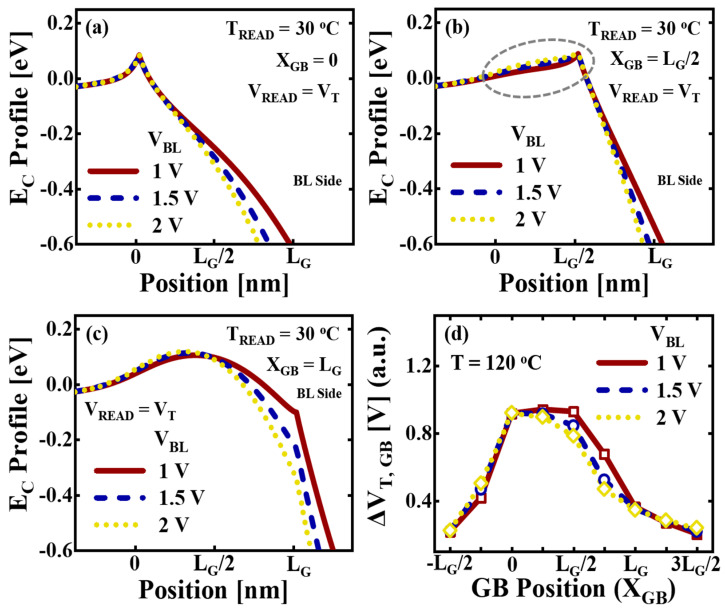
The simulated *E_C_* profiles at the BE-TOX/channel interface along the BL direction at *T_READ_* = 30 °C. The *V_READ_* is fixed as *V_T_*, and the *V_BL_* varies with 1 V, 1.5 V, and 2 V. The GB under the target cell is located at (**a**) *X_GB_* = 0, (**b**) *X_GB_* = *L_G_*/2, and (**c**) *X_GB_* = *L_G_*. (**d**) The simulated Δ*V_T,GB_* depending on each *X_GB_* ranging from -*L_G_*/2 to 3*L_G_*/2 by *V_BL_* (= 1 V, 1.5 V, 2 V) for *T* = 120 °C.

Figure 9a shows the simulated *E_C_* profiles of the channel depending on the *V_BL_* at *T_READ_* = 30 °C for *X_GB_* = 0. The *Φ_B_* remains consistent regardless of the *V_BL_*. Figure 9b shows the *E_C_* profiles for *X_GB_* = *L_G_*/2. As the *V_BL_* increases, the *E_C_* peak induced by the word line voltage difference between the target cell and adjacent cells shifts towards the SL side. For this reason, the additional barrier effect induced by the *Φ_B_* of GB is diminished. The *E_C_* near the SL side becomes higher and the influence of GB decreases with the increase in *V_BL_*. Figure 9c shows the *E_C_* profiles for *X_GB_* = *L_G_*. As the *V_BL_* increases, the *E_C_* peak induced by the word line voltage difference shifts towards the SL side. The GB effect, which is nearly negligible at *V_BL_* = 1 V, indicates no variation with respect to *V_BL_*. Figure 9d shows the simulated Δ*V_T,GB_* depending on *V_BL_* (= 1 V, 1.5 V, and 2 V) for *T* = 120 °C at each *X_GB_*. When the GB is located near the SL side edge under the target cell, the Δ*V_T,GB_* is less influenced by *V_BL_*. However, as the *X_GB_* shifts from 0 to *L_G_*, the Δ*V_T,GB_* decreases due to the elevated *V_BL_*. At the *X_GB_* under the spacer region, where the influence of GB is already limited, the variation in Δ*V_T,GB_* caused by *V_BL_* is not substantial. Therefore, the average Δ*V_T,GB_* decreases with the increase in *V_BL_*, as shown in Figure 7b.

## 3. Conclusions

The retention characteristics in HPLR conditions are investigated to understand the cross-temperature effects on the *V_T_* variation in 3-D NAND flash memory. The HPLR conditions consisting of the *T_PGM_* with a range of 30 °C to 120 °C and *T_READ_* of 30 °C are used. The Δ*V_T_* of the programmed cell is successfully decomposed into the Δ*V_T,CL_* and the Δ*V_T,GB_* using a neutral cell. The Δ*V_T,GB_* is lower than the Δ*V_T,CL_* at *T_PGM_* = 75 °C. Compared to the Δ*V_T,CL_*, the Δ*V_T,GB_* becomes dominant at *T_PGM_* = 120 °C due to the decrease in *Φ_B_* of the GB. The average Δ*V_T,GB_* extracted from the 50 cells decreases by 10% with increasing *V_BL_* from 1 V to 2 V. The effect of *V_BL_* on Δ*V_T,GB_* varies according to the *X_GB_*. The characteristics of Δ*V_T,GB_* versus *V_BL_* can be utilized to measure the GB’s influences on the characteristics of *I_BL_* in 3-D NAND flash memory.

## Data Availability

The data presented in this study are available on request from the corresponding author. The data are not publicly available due to confidentiality request.
